# Chemoselective transfer hydrogenation of aldehydes in aqueous media catalyzed by a well-defined iron(II) hydride complex

**DOI:** 10.1007/s00706-018-2279-7

**Published:** 2018-10-19

**Authors:** Nikolaus Gorgas, Aleksandra Ilic, Karl Kirchner

**Affiliations:** Institute of Applied Synthetic Chemistry, Vienna University of Technology, Getreidemarkt 9/163-AC, 1060 Vienna, Austria

**Keywords:** Aldehydes, Transfer hydrogenation, Chemoselective reduction, Aqueous media, Iron catalyst

## Abstract

**Abstract:**

An iron(II) hydride PNP pincer complex is applied as catalyst for the chemoselective transfer hydrogenation of aldehydes using an aqueous solution of sodium formate as hydrogen source. A variety of aromatic, heteroaromatic, and aliphatic aldehydes could be reduced to the corresponding alcohols in good to excellent yields with a catalyst loading of 1.0 mol% at 80 °C and 1 h reaction time. If present, C–C double bonds remained unaffected in course of the reaction, even when they are conjugated to the carbonyl group of the aldehyde. The catalyst’s lifetime and activity could be improved when the reactions were conducted in an ionic liquid-based micro emulsion.

**Graphical abstract:**

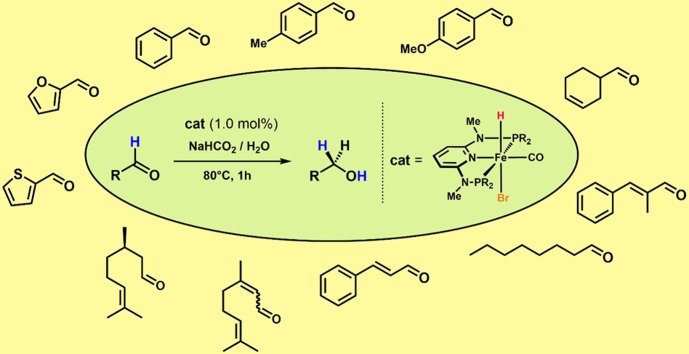

## Introduction

The reduction of aldehydes represents an important industrial process since the respective primary alcohols are required in large amounts for the production of flavors, frequencies, and pharmaceuticals [1–4]. As the cleanest and most atom-economical approach, transition-metal catalyzed hydrogenation is mainly employed for this purpose. However, the need for high-pressure equipment and concerns about safety issues limit the applicability of this method. The use of hazardous hydrogen gas can be avoided by employing related transfer hydrogenation reactions that provide a safe and operationally simple alternative [5]. The use of formic acid or formate salts as reductant is particularly attractive since water can be used as reaction medium providing access to a green and sustainable process. Several iridium- and ruthenium-based catalysts have been reported to excellently promote the selective transfer hydrogenation of aldehydes using water as solvent and formates as hydrogen source [6–9, 10].

Within this context, our group developed iron(II) PNP pincer complexes over the past years that are capable for the hydrogenation and dehydrogenation of carbonyl compounds [11, 12]. For example, the hydrido carbonyl complex **1** was found to efficiently promote the chemoselective hydrogenation of aldehydes exhibiting unprecedented activity and productivity under mild conditions (Scheme 1) [13, 14]. The same catalyst could successfully be employed in the production of dihydrogen by catalytic decomposition of formic acid [15]. In both cases, the iron(II) dihydride species **2** could be identified as the catalytically active species. Therefore, it appeared obvious to combine these two processes to reduce carbonyl compounds employing formic acid (FA) as hydrogen donor. In the present paper, we now report on the chemoselective reduction of aldehydes under transfer hydrogenation conditions using water as reaction medium.

**Figure:**
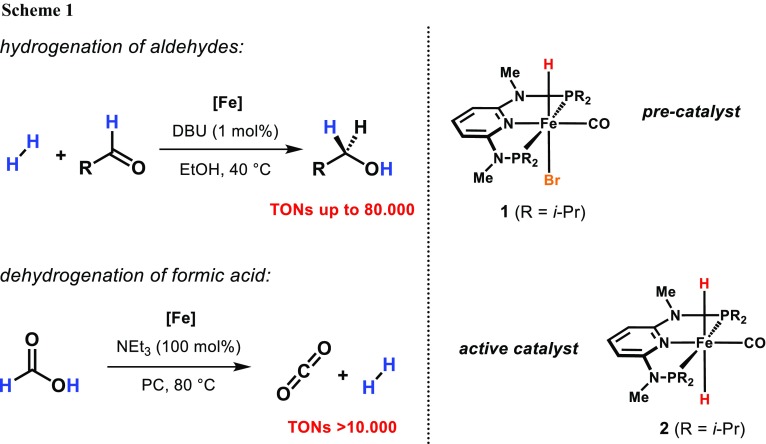

## Results and discussion

To validate our concept, preliminary experiments were performed under similar conditions as previously reported for FA dehydrogenation (THF, 40–60 °C) using 1.0 mol% of catalyst, 4-fluorobenzaldehyde as substrate (2.0 mmol), and a 1:1 mixture of FA/NEt_3_ as reductant (10 mmol). However, only marginal conversion into the respective primary alcohol could be detected by ^19^F{^1^H} NMR spectroscopy. Instead, we observed strong gas evolution and complete consumption of formic acid indicating that H_2_ formation takes place much faster than hydrogen transfer to the substrate, since the intermediately formed dihydride species presumably gets protonated before it can react with an aldehyde carbonyl group.

To circumvent this problem, we envisaged the use of an aqueous sodium formate solution as reducing agent, which was added to solutions of the catalyst and the substrate in different organic solvents. First, the experiments were conducted at 80 °C for 1 h and the outcome of these reactions is summarized in Table 1. Depending on the co-solvent, conversions between 33 and 84% were obtained but best results could be achieved when the aqueous solution was combined with a neat mixture of the catalyst and the substrate giving full conversion within 1 h. The catalytic activity significantly dropped when the reaction temperature was lowered to 60 °C. Decreasing the catalyst loading to 0.5 and 0.25 mol% resulted in 74 and 53% yield, respectively, which corresponds to TOFs of more than 200 h^−1^. Only slightly higher conversions were achieved by extending the reaction time indicating insufficient long term stability of the catalyst.

**Table 1:** Transfer hydrogenation catalyzed by **1** using NaHCO_2_/H_2_O as reductant
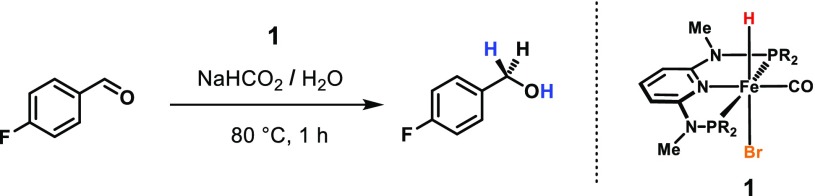

Entry	Cat. loading/mol%	Solvent	Yield/%^a^
1	1.0	1,4-dioxane	46
2	1.0	Heptane	33
3	1.0	EtoH	46
4	1.0	Toluene	84
5^b^	1.0	Neat	56
6	1.0	Neat	> 99
7	0.5	Neat	74
8	0.25	Neat	52
9^c^	0.25	Neat	65

Conditions: subst. (2.0 mmol), cat., NaHCO_2_ (2.5 mmol/2.5 M in H_2_O), co-solvent (1.0 cm^3^); 80 °C, 1 h

^a^Determined by ^19^F{^1^H}NMR

^b^Performed at 60 °C

^c^17 h reaction time

Thus, we investigated the general applicability of this protocol by testing the reduction of various substrates in presence of 1.0 mol% of **1** applying a reaction time of 1 h. Gratifyingly, a variety of aromatic, heteroaromatic, and aliphatic aldehydes could be reduced giving the corresponding alcohols in good to excellent yields. If present, C–C double bonds remained unaffected in course of the reaction, even when they are conjugated to the carbonyl group of the aldehyde as in the case of some industrial important substrates such as citral (**A8**) or cinnamaldehyde (**A9**). Moreover, ketones are not reduced by **2** as demonstrated by a test reaction employing 4-fluoroacetophenone as substrate (Table 2).

**Table 2:**
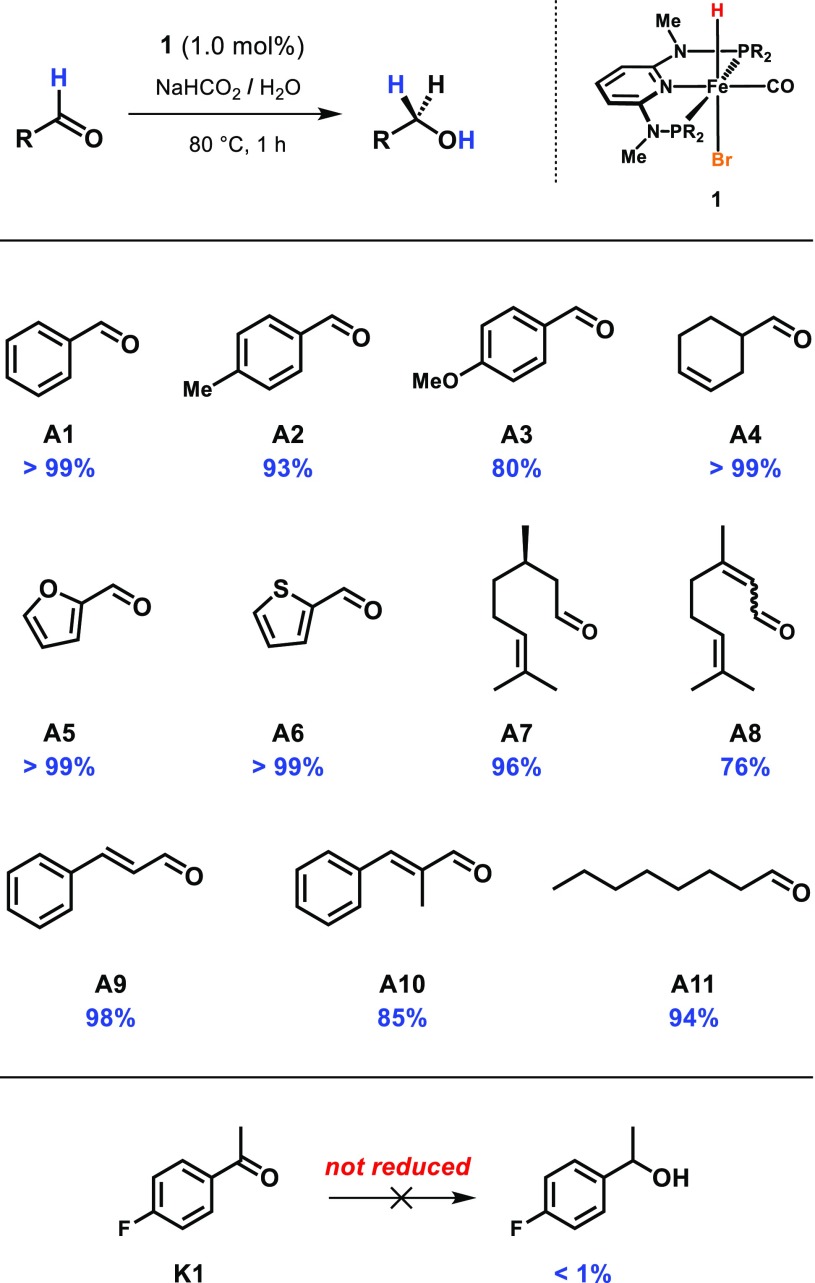
Substrate screening using pre-catalyst **1** under transfer hydrogenation condition (yields determined by ^1^H NMR)

**Fig. 1:**
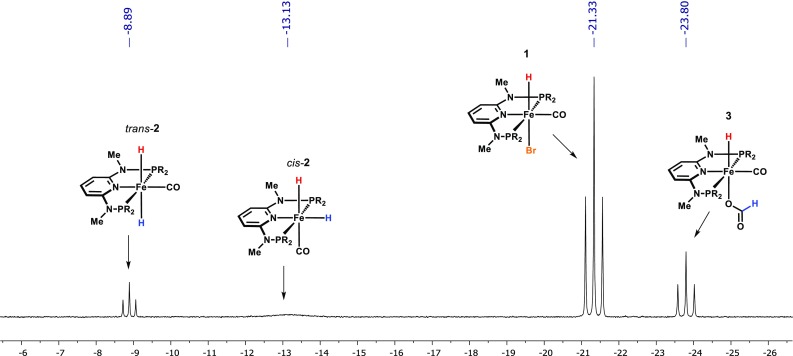
^1^H NMR spectrum (hydride region) recorded during mechanistic studies

From a mechanistic perspective, the iron dihydride complex **2** is supposed to represent the catalytically active species in the reaction. To verify our assumption, an NMR experiment has been conducted in which a biphasic mixture of **1** in toluene-*d*_*8*_ and an aqueous solution of sodium formate was heated up to 80 °C for 20 min. NMR analysis of the toluene phase revealed the formation of the anticipated iron(II) dihydride species **2** which is present as a mixture of a *cis* and *trans* isomer [14]. The triplet resonance at − 23.80 ppm shown in Fig. 1 can be assigned to the hydride formate complex **A** [16]. The major species, however, still remains the pre-catalyst **1** pointing towards incomplete catalyst activation. However, test reactions using the isolated iron dihydride **2** gave almost equal results as obtained with pre-catalyst **1**.

Based on our previous studies [13–16] as well as the observations made in the NMR experiment described above, a catalytic cycle has been proposed which is depicted in simplified form in Scheme 2. The pre-catalyst **1** gets activated by replacement of the bromide ligand by the formate anion. This intermediate is prone to undergo a non-classical β-hydride elimination resulting in the formation of the iron(II) dihydride species **2** and liberation of carbon dioxide. Due to its strong hydridic character [17], *trans*-**2** (**B**) is capable of a direct nucleophilic attack on the carbonyl carbon atom of the aldehyde substrate to form the alkoxide complex **C**. This intermediate is then protonated by H_2_O to give the final alcohol. Substrate release provides a vacant coordination site (**D**) allowing another formate molecule to bind on to the metal center to close the catalytic cycle.

**Figure:**
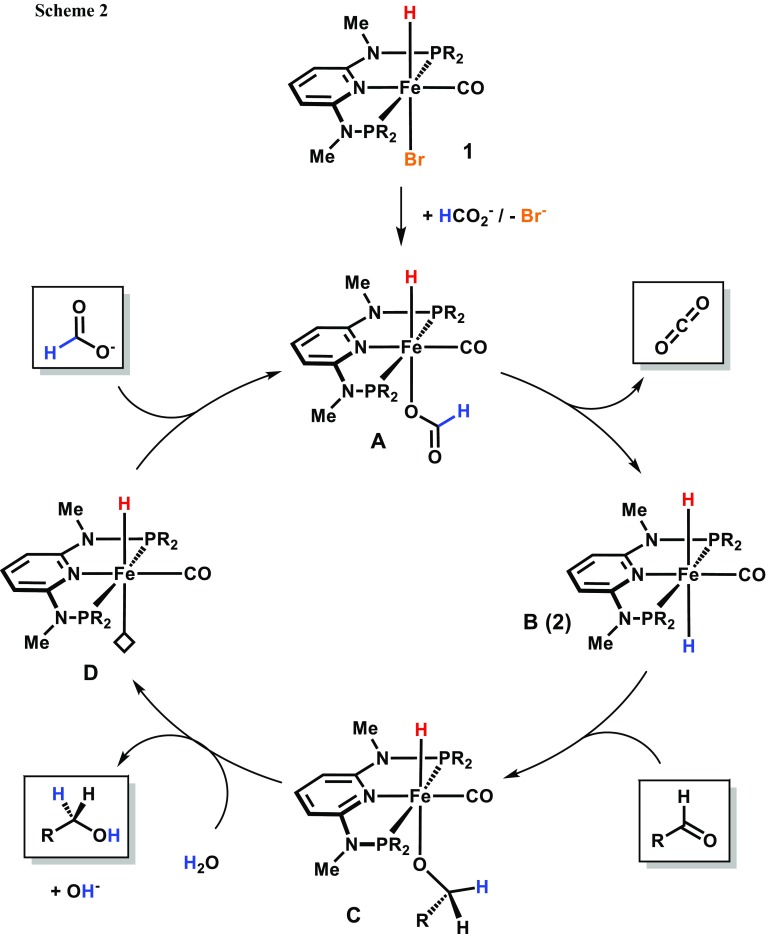

**Fig. 2:**
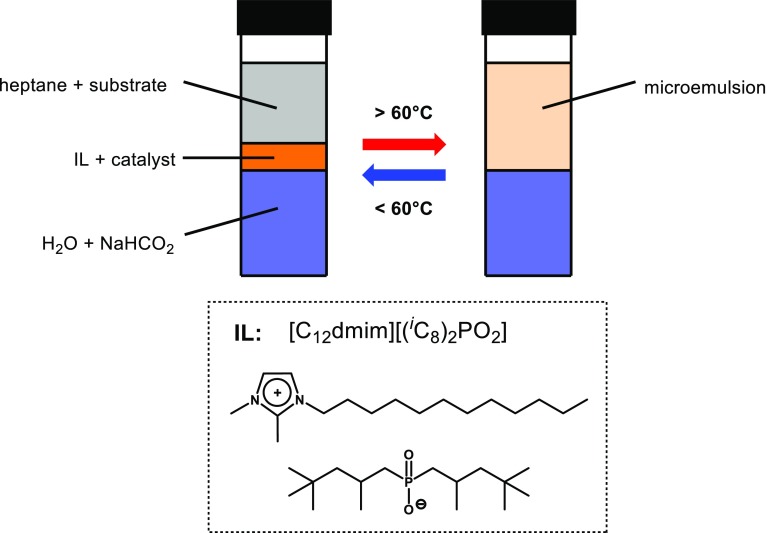
Hydrogenation under three-phasic conditions

Since catalyst **1** was recently reported to be highly compatible with ionic liquids (IL) [18], we tried to improve the catalyst lifetime and activity by conducting the iron-catalyzed transfer hydrogenation in ionic liquid-based micro emulsions. In particular, it appeared promising to apply a three-phasic system consisting of the aqueous formate solution, a heptane phase containing the substrate and the catalyst dissolved in a separate phase of the ionic liquid [C_12_dmim][(^i^C_8_)_2_PO_2_] (Fig. 2). Such a system, as recently applied in palladium-catalyzed cross-coupling reactions, is capable of forming a stable micro emulsion at elevated temperatures which, after cooling, again splits in separate phases and thus facilitates product separation and catalyst recycling [19].

In initial experiments, the catalytic activity could significantly be enhanced giving full conversion of 4-fluorobenzaldehyde within 1 h even at 60 °C and a catalyst loading of 0.5 mol%. However, after cooling to room temperature, the reaction mixture did not separate which might be caused by the comparatively high polarity of the product alcohol. Further attempts by varying the relative ratio of the aqueous, heptane, and IL-phase failed to achieve phase separation.

Nevertheless, the increased catalytic activity might be attributed to the fact that the ionic liquid facilitates the mutual phase transfer of substrate and the reductant. Therefore, we employed [C_12_dmim][(^i^C_8_)_2_PO_2_] as surfactant in the reaction. The results summarized in Table 3 clearly show a beneficial effect on the catalytic activity when the reaction is carried out in the presence 10 mol% of the ionic liquid as turnover frequencies were increased by an order of magnitude. Moreover, the catalyst also proved to be more stable under these conditions reaching turnover numbers of more than 700 within 18 h.

**Table 3:** Transfer hydrogenation catalyzed by **1** using NaHCO_2_/H_2_O as reductant in the presence of ionic liquid [C_12_dmim][(^i^C_8_)_2_PO_2_]
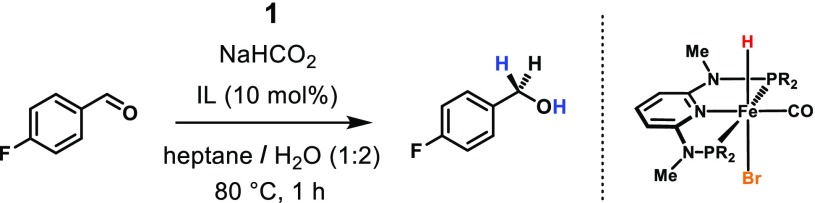

Entry	Cat. loading/mol%	*t*/*h*	Yield/%^a^
1^b^	1.0	1	33
3	0.5	1	96
3	0.2	1	66
4	0.1	1	37
5	0.5	18	> 99
6	0.2	18	95
7	0.1	18	74

Conditions: subst. (2.0 mmol), cat., NaHCO_2_ (2.5 mmol/2.5 M in H_2_O), ionic liquid (10 mol%), co-solvent (0.5 cm^3^); 80 °C, 1 h

^a^Determined by ^19^F{^1^H}NMR

^b^Carried out without ionic liquid

## Conclusion

In summary, we presented a proof-of-principle study demonstrating that the iron(II) hydride complex **1** can be applied as catalyst for the chemoselective transfer hydrogenation of aldehydes using an aqueous solution of sodium formate as hydrogen source. A variety of aromatic, heteroaromatic, and aliphatic aldehydes could be reduced to the corresponding alcohols in good to excellent yields. If present, C–C double bonds remained unaffected in course of the reaction, even when they are conjugated to the carbonyl group of the aldehyde. To improve the catalyst lifetime and activity, the transfer hydrogenation reactions were conducted in an ionic liquid-based micro emulsion. When the reaction is carried out in the presence of 10 mol% of ionic liquid, turnover frequencies increased by an order of magnitude.

## Experimental

All manipulations were performed under an inert atmosphere of argon using Schlenk techniques or in a MBraun inert-gas glovebox. Deuterated solvents were purchased from Euriso-top and dried over 4 Å molecular sieves. All aldehyde substrates were obtained from commercial sources and distilled prior to use. Complexes **1** and **2** were prepared according to literature procedures [13, 14]. ^1^H, ^13^C{^1^H}, and ^31^P{^1^H} NMR spectra were recorded on a Bruker AVANCE-250 spectrometer. ^1^H and ^13^C{^1^H} NMR spectra were referenced internally to residual protio-solvent and solvent resonances, respectively, and are reported relative to tetramethylsilane (*δ* = 0 ppm). ^31^P{^1^H} NMR spectra were referenced externally to H_3_PO_4_ (85%) (*δ* = 0 ppm).

### General procedure for the catalytic transfer hydrogenation of aldehydes

In a typical experiment, a vial containing a magnetic stirring bar was charged with catalyst **1** and the substrate (2.0 mmol) inside a glovebox. The vial was sealed with a septum screw cap, taken out from the glovebox and a solution of sodium formate in degassed water (1.0 cm^3^, 2.5 M) was added through the septum. The reaction mixture was stirred at 80 °C for the specified time after which it was quickly cooled to room temperature and the reaction was quenched by exposure to air. A sample was taken from the organic phase, diluted in CDCl_3_, and analyzed by NMR spectroscopy. For the isolation of the reaction products, 1 cm^3^ diethyl ether was added and the phases were separated. The aqueous phase was washed with diethyl ether and the combined extracts were filtered over a short plug of silica to remove the catalyst. The solution was dried over MgSO_4_ and the solvent removed under reduced pressure.
